# Supplementary material for: Chromothripsis during telomere crisis is independent of NHEJ, and consistent with a replicative origin
Source: Genome Res. 2019 May;29(5):737–49. doi: 10.1101/gr.240705.118 (PMC6499312; doi:10.1101/gr.240705.118)
Supplement: Supplemental Material [file supp_gr.240705.118_Supplemental_file_1.zip › contigs/annotated_contigs/DB113/contig.2.DB113_length_587_mean_cov_18.262350937.docx]

**DB113_length_587_mean_cov_18.262350937**

```
GGCTCACACCTGCCATCCCAGGACTTTGAGAGGTTGAGGCGGGTGGATCACTTGAGGTCAAAAGTACGAGGCTGGCCTGGCCAACATGT
 >chr19:53956860-53957229 + E=2e-204
TGAAACCCTATCTCTACTAAAAATACAAAAATTAGCCAAGCATGGTGGTGTGTGCCTGTAATCCCAGGTACTCAGGAGGCTGAGGCAGG

AGAATGGCTTCAACCCAAAAGGTGGATGTTGCAGTGAGCCGAGATCAGGCCATTGAACTCCAGCCAGTTGCAACAGAGTGAAACTTCAT

CTCAAAAATATAAAAATAAAAAAAATGTTTTAATGTCACAACGTGCCTTTTCTTCATGGATATAGGTAATTTTATGACAGTTATTCAGA

AAAACATTGTAT|T|CCGGCTAAAGGTCTTTCCACACTCATTACACTTGTAAGCATTGTCTTCAGTATGAATTTTCCGATGTATTTCAA
             >chr19:53958890-53959109 - E=5e-111
AATTTGATCTGAAACTGTAAGCTTTGTCACATTCTTCACATTTGTAAGGTTTCTCTCCAGTATGAAGTCTACGATGGCATGTAAGGGAT

GACTTCTGACTAAAGGTCTTGCCACACTCATTACACTTGTAAGGTTTCTCTCCAG
```
